# Real-Time Visualization of the Mascagni-Sappey Pathway Utilizing ICG Lymphography

**DOI:** 10.3390/cancers12051195

**Published:** 2020-05-08

**Authors:** Anna Rose Johnson, Melisa D. Granoff, Hiroo Suami, Bernard T. Lee, Dhruv Singhal

**Affiliations:** 1 Division of Plastic and Reconstructive Surgery, Beth Israel Deaconess Medical Center, Harvard Medical School, Boston, MA 02215, USA; anna.johnson719@gmail.com (A.R.J.); melisagranoff@gmail.com (M.D.G.); blee3@bidmc.harvard.edu (B.T.L.); 2 Faculty of Medicine and Health Sciences, Macquarie University, Sydney 2109, NSW, Australia; hiroo.suami@mq.edu.au

**Keywords:** M-S pathway, lymphatic anatomy, BCRL

## Abstract

Background: Anatomic variations in lymphatic drainage pathways of the upper arm may have an important role in the pathophysiology of lymphedema development. The Mascagni–Sappey (M–S) pathway, initially described in 1787 by Mascagni and then again in 1874 by Sappey, is a lymphatic drainage pathway of the upper arm that normally bypasses the axilla. Utilizing modern lymphatic imaging modalities, there is an opportunity to better visualize this pathway and its potential clinical implications. Methods: A retrospective review of preoperative indocyanine green (ICG) lymphangiograms of consecutive node-positive breast cancer patients undergoing nodal resection was performed. Lymphography targeted the M-S pathway with an ICG injection over the cephalic vein in the lateral upper arm. Results: In our experience, the M-S pathway was not visualized in 22% (*n* = 5) of patients. In the 78% (*n* = 18) of patients where the pathway was visualized, the most frequent anatomic destination of the channel was the deltopectoral groove in 83% of patients and the axilla in the remaining 17%. Conclusion: Our study supports that ICG injections over the cephalic vein reliably visualizes the M-S pathway when present. Further study to characterize this pathway may help elucidate its potential role in the prevention or development of upper extremity lymphedema.

## 1. Introduction

Our current understanding of the development of breast cancer-related lymphedema (BCRL) is largely based on established risk factors including axillary lymph node dissection (ALND), regional lymph node radiation (RLNR), and elevated body mass index (BMI) [1,2,3]. Current data supports that breast cancer patients with the top two risk factors of ALND and RLNR have only a 33% incidence of lymphedema [4]. There is no current consensus on why the remainder of high-risk patients (67%) undergoing ALND and RLNR do not develop lymphedema. One theory that may account for this is the presence of anatomic variations in lymphatic drainage that could predispose, or protect, an individual from the development of lymphedema. Specifically, an alternate lymphatic pathway that drains the arm and bypasses the axilla was described in 1787 by Mascagni and again in 1874 by Sappey and has been termed the Mascagni–Sappey (M–S) pathway [5,6]. The presence or absence of the M–S pathway has been postulated to contribute to an individual’s risk of developing lymphedema. However, visualization or imaging of any lymphatic pathway has been a daunting challenge, as this part of the vascular system is one logarithmic scale smaller than arteries or veins [7]. Nonetheless, developing a technique to reliably visualize the M–S pathway in vivo would be an important steppingstone to uncovering possible individual anatomic variations in lymphatic anatomy that may ultimately shed light on which patients are most prone to the development of breast cancer-related lymphedema. 

Early attempts to map the lymphatic system can be traced to the 17th century with the use of mercury [8]. Efforts to visualize the M–S pathway can be traced back to the 18th century and were largely based on anatomic dissections [5,6]. This accessory lymphatic pathway was first described by Mascagni in cadaveric specimens and then again by Sappey in the 19th century. Additional work by anatomists Kubik and Leduc clarified the presence of this pathway in the lateral upper arm and charted its anatomic course alongside the cephalic vein [9,10]. Kubik reported that the M–S pathway, when present, had variable anatomic destinations, including the axilla and the supraclavicular nodes [10]. In more modern work, Tashiro et al. used indocyanine green (ICG) to visualize the upper arm lymphatic drainage patterns of breast cancer patients with lymphedema [11]. They found that the presence of accessory drainage pathways which terminated outside of the axilla were associated with a less severe presentation of lymphedema. Akita et al. used ICG imaging as a modality for the early detection of lymphedema. In this study, they noted the presence of collateral drainage pathways in breast cancer patients who developed lymphatic dysfunction and then improved [12]. Prior studies which have localized accessory pathways are limited, as they were either performed in cadaveric specimens or in patients with lymphedema [5,6,9,11,12]. In patients with lymphedema, the ability to visualize distinct channels in the upper extremity is altered. Additionally, accessory pathways visualized in patients with BCRL may have been present preoperatively or developed later via collateral drainage routes. 

These studies emphasize the need to prospectively investigate and explore the M–S pathway as a compensatory drainage pathway for lymphedema prevention. Our study specifically targeted and mapped the M–S pathway in preoperative patients without lymphedema utilizing ultrasound-guided ICG injections over the cephalic vein. We contend that an improved knowledge of anatomic variations in upper arm drainage patterns in a patient cohort without lymphedema may ultimately improve our understanding of the pathophysiology of BCRL development, and guide preoperative, intraoperative, and postoperative management.

## 2. Results

Our review identified 23 consecutive patients with newly diagnosed, lymph node-positive unilateral breast cancer undergoing preoperative ICG lymphography before surgical oncologic management. Cancer staging of each patient can be found in Table S1. All patients were female with a mean age of 51.6 years and an average BMI of 28.2 kg/m^2^. The majority of patients were Caucasian (74%) and reported their ethnicity as non-Hispanic (83%). All patients (100%) had confirmed lymph node-positive disease. Additional characteristics can be found in Table 1. Of the 57% of patients who underwent neoadjuvant chemotherapy, 92% received taxane-based regimens. The median number of nodes removed during ALND was 15. Of these, a median of one node was positive. 

**Figure 1:**
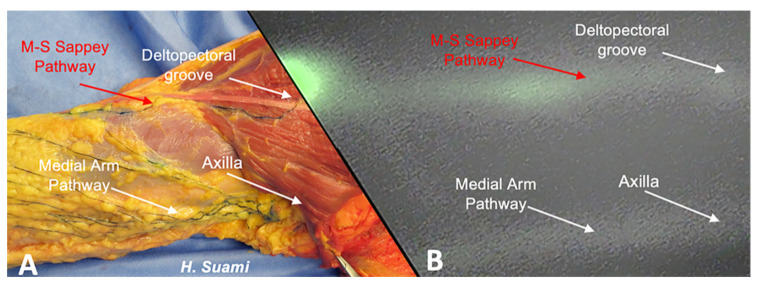
Cadaveric dissection and indocyanine green (ICG) findings demonstrating the presence of the Mascagni–Sappey (M–S) pathway and a distinct medial arm pathway. (**A**) Cadaveric dissection illustrating the M-S pathway terminating in the deltopectoral groove and a distinct medial arm pathway terminating in the axilla. (**B**) ICG imaging of an upper extremity in vivo demonstrating the M–S pathway terminating in the deltopectoral groove and a distinct medial arm pathway terminating in the axilla.

**Figure 2:**
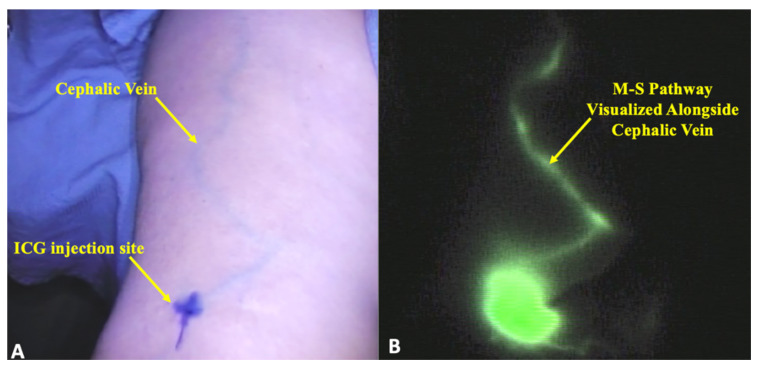
M–S pathway visualized coursing along the cephalic vein. (**A**) Location of ICG injection over the cephalic vein 4 cm proximal to the antecubital crease. The cephalic vein course can be grossly visualized in the upper arm. (**B**) M–S pathway visualized coursing along the cephalic vein utilizing ICG imaging.

Our injection and imaging technique provided comprehensive visualization of the draining lymphatics of the upper arm. Linear lymphatic channels were universally identified in all patients arising from the hand and volar forearm injections. Following the ultrasound-guided injections over the cephalic vein, five patients (22%) demonstrated no lymphatics in the lateral arm. In 18 patients (78%), a lymphatic channel of the lateral upper arm, or M-S pathway, was noted to course alongside the cephalic vein (Figure 1 and Figure 2). In these patients, the most common anatomic destination of the M–S pathway into the torso was the deltopectoral groove in 83% of patients (*n* = 15), followed by the axilla in 17% of patients (*n* = 3).

## 3. Discussion

In this study, we reliably identified the Mascagni–Sappey pathway using a novel ICG lymphography protocol, which involved an ultrasound-guided injection of ICG directly over the cephalic vein. Moreover, using this technique, we were able to identify the M–S pathway in 78% of patients. When visualized, the M–S pathway was able to be visualized to the deltopectoral groove (83%) or the axilla (17%).

Our novel ICG lymphography technique involves intradermal injection of ICG over the cephalic vein. Our findings are consistent with lessons learned during prior cadaveric anatomic dissections, where, when present, the M-S pathway was noted to be located alongside the course of the cephalic vein [9]. Our in vivo data are consistent with findings from anatomic dissections where Leduc et al. identified the M–S pathway in 76% of 300 cadaveric dissections [13]. This supports the reliability of this technique for detection of the M–S pathway in patients without lymphatic dysfunction. Moreover, we noted anatomic variations in the course of the M–S pathway. The pathway predominately drained to the deltopectoral groove; however, in a minority of patients the lateral pathway was found draining to the axilla. We have utilized these findings to modify our approach to immediate lymphatic reconstruction (performing a lymphovenous bypass at the time of axillary lymph node dissection). Specifically, at our institution, we use a blue dye injection over the cephalic vein to map the lateral lymphosome versus fluorescein over the medial lymphosome. Intraoperatively, if the M–S pathway (blue dye) is noted to be draining into the axilla, we will bypass the lymphatics from this distinct lymphosome, along with those from the main pathway from the medial upper arm (fluorescein dye) [14].

Ultimately, the fundamental question as to why 67% of patients with the top two risk factors (ALND and regional lymph node radiation) for lymphedema do not go on to develop BCRL remains unclear. We believe the presence of the M–S pathway and its anatomic course are central to answering this question. Suami et al. proposed that the M–S pathway may function as a detour route when the axillary (main) pathway is damaged [15]. Pissas et al. underscored the intraoperative need to protect these secondary pathways, including the lateral arm M-S pathway, as it may be an essential shunt for patients undergoing nodal extirpation of the axilla [16]. Turfe et al. described a case where potential disruption of the M–S pathway via placement of a chemotherapy port resulted in BCRL. The authors posit that the port placement disrupted the only functional upper arm pathway remaining after ALND [13]. The ability to reliably detect this pathway has important implications for patient management and surveillance. Patients without this compensatory pathway, or those with known iatrogenic surgical damage or targeted radiotherapy to this pathway, may require more aggressive surveillance for clinical signs and symptoms of lymphedema. Moreover, the presence or absence of this pathway may better inform those who would best benefit from immediate lymphatic reconstruction. While there is some evidence in the literature that neoadjuvant chemotherapy may impact lymphatic function, our cohort is underpowered to draw any conclusions regarding its effect on visualization of the M–S pathway [17]. Further, it is worthwhile to consider if our findings can be generalized to the greater population. Specifically, our findings represent those of women with node-positive disease and evidence of metastatic breast cancer to the axilla. However, overall, a median of one in 15 removed nodes indeed demonstrated metastatic disease presence. While performing this study on a healthy patient cohort would be ideal, it is unlikely that the small burden of nodal disease in the axilla (median 1 in 15 nodes positive) affected our ICG findings. We look forward to future studies to further evaluate the M–S pathway and its relationship to lymphedema development and severity. Nonetheless, the key to further study on this topic fundamentally requires reliable visualization of this pathway and its course.

This study presents a unique approach to visualizing lymphatic drainage anatomy of the arm and its variability, using ICG lymphography, with targeted injections to identify the M-S pathway. We acknowledge that the M–S pathway can sometimes be visualized from hand and forearm injections alone; however, a targeted injection over the cephalic vein in the upper arm provides a comprehensive methodology to ensure that the M–S pathway, when present, can be visualized. We believe continued investigation of the anatomic variability in the lateral pathway of the upper arm has the potential to provide important insights into the pathophysiology of BCRL development.

## 4. Materials and Methods

A retrospective review of consecutive newly diagnosed breast cancer patients undergoing preoperative ICG lymphography between October 2018 and March 2019 was performed. All patients eligible for ICG lymphography were scheduled for ALND and had lymph node-positive disease. All patients had previously undergone either sentinel lymph node biopsy (SLNB) or fine needle aspiration (FNA) for diagnosis of their node-positive disease prior to surgery (Table 1). Institutional Review Board approval was obtained for this study (#2019-P-000-047). Patient demographics, clinical characteristics, and ICG lymphography reports and images were retrieved and entered into a study-specific REDCap database [18].

### Injection Technique

**Figure 3:**
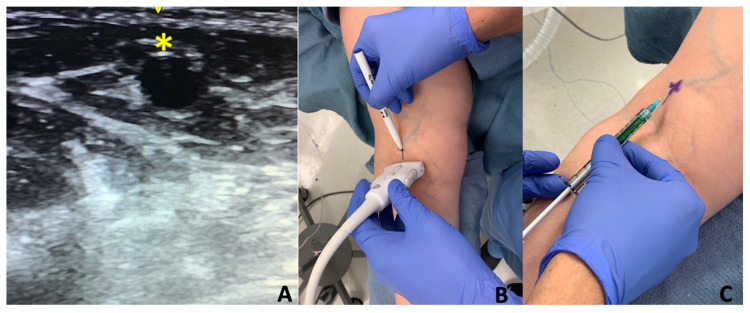
Preoperative ultrasound (US) localization over the cephalic vein. (**A**) The asterisk (*) demarcates the location of the cephalic vein using ultrasound technology. (**B**) Depiction of the operating surgeon mapping out the injection point 4 cm distal to the antecubital crease over the cephalic vein. (**C**) Targeted injection of ICG and albumin mixture to map the lymphatic anatomy of the upper extremity.

Under sterile conditions, 0.1 cc of stock (0.625 mg/cc) ICG solution (Akorn Inc., Lake Forest, IL, USA) with albumin was injected intradermally at four anatomic locations: 1 cm proximal to the first and fourth web spaces, 1 cm proximal to the wrist crease in the volar forearm, and 4 cm proximal to the antecubital crease immediately over the cephalic vein, which was identified by ultrasound (Figure 3). A near-infrared (NIR) imaging device, the PDE-Neo II (Hamamatsu Photonics KK, Hamamatsu, Japan), was used to visualize patterns of superficial lymphatic drainage of the operative extremity. A single surgeon (D.S.) performed and interpreted all consecutive ICG lymphangiograms. Two independent researchers (A.R.J., M.D.G.) analyzed all the data.

## 5. Conclusions

In this study, we introduce a novel injection and imaging technique as a reliable way to visualize the M–S pathway in patients without upper extremity lymphedema. In our cohort, the M–S pathway was present in the majority, but not all patients. Although the M-S pathway was noted to drain to the deltopectoral groove in the majority of patients, it was also found to infrequently terminate in the axilla, highlighting its variability. This historically understudied pathway warrants attention as it may provide critical insight into why certain patients may be predisposed to or protected from lymphedema development.

## Figures and Tables

**Table 1:** Patient demographics.

Demographics and Cancer Treatment Characteristics *n* = 23	
Age, mean (SD ^^^)	51.6 (17)
BMI, kg/m2, mean (SD)	28.2 (6)
Race, *n* (%)	
Caucasian	17 (74)
Black/African American	4 (17)
Asian	2 (9)
Ethnicity, *n* (%)	
Hispanic	4 (17)
Non-Hispanic	19 (83)
Neoadjuvant chemotherapy, *n* (%)	13 (57)
Taxane-based, *n* (%)	12 (92)
Axillary intervention	
Sentinel lymph node biopsy (SLNB) ^†^, *n* (%)	14 (61)
Nodes removed in SLNB, median (IQR *)	4 (2–5)
Axillary lymph node dissection (ALND) ^‡^, *n* (%)	23 (100)
Positive nodes removed, median (IQR)	1 (0–3)
Total nodes removed in ALND, median (IQR)	15 (9–22)

^ SD: Standard deviation; * IQR: inter-quartile range; † All SLNB were performed in a staged manner prior to ICG lymphography; ‡ all ALND were performed after ICG lymphography.

## Supplementary Materials

The following are available online at https://www.mdpi.com/2072-6694/12/5/1195/s1, Table S1: Patient Cohort Cancer Stage.
