# Supplementary material for: Real-Time Visualization of the Mascagni-Sappey Pathway Utilizing ICG Lymphography
Source: Cancers (Basel). 2020 May 8;12(5):1195. doi: 10.3390/cancers12051195 (PMC7281680; doi:10.3390/cancers12051195)
Supplement: Supplementary file 1 [file cancers-12-01195-s001.pdf]

Supplementary Materials

Real-Time Visualization of the Mascagni-Sappey Pathway Utilizing ICG Lymphography

Anna Rose Johnson, Melisa D. Granoff, Hiroo Suami, Bernard T. Lee and Dhruv Singhal

Table S1. Patient Cohort Cancer Stage.

**Patient Cohort Cancer Stage** ***n = 23***	
**Cancer Stage**	**n^ (%)**
IA	2 (9)
IIA	6 (26)
IIB	7 (30)
IIIA	6 (26)
IIIB	1 (4)
IIIC	1 (4)

n^ = number of patients

© 2020 by the authors. Licensee MDPI, Basel, Switzerland. This article is an open access article distributed under the terms and conditions of the Creative Commons Attribution (CC BY) license (http://creativecommons.org/licenses/by/4.0/).
